# Inner engineering is associated with perceived improvements in relationship quality, interpersonal connections, and interpersonal compassion

**DOI:** 10.3389/fpsyg.2024.1454293

**Published:** 2024-12-23

**Authors:** Nashaw Jafari, Atina Manvelian, Enas Mohamed, Akila Rayapuraju, Hibiki Orui, Sultan Alkuhaimi, Preeti Upadhyay Reed, Balachundhar Subramaniam

**Affiliations:** ^1^Sadhguru Center for a Conscious Planet, Beth Israel Deaconess Medical Center, Harvard Medical School, Boston, MA, United States; ^2^Counseling Psychology, Santa Clara University, Santa Clara, CA, United States

**Keywords:** interpersonal mindfulness, relationship quality, mindfulness-based practices, wellbeing, perceived stress, compassion

## Abstract

**Objectives:**

Interpersonal relationships are a cornerstone of wellbeing. Mindfulness-based practices have been shown to improve relationship quality and reduce perceived stress. Inner Engineering (IE) is a transformative program that includes meditative and yogic practices associated with improvements in mindfulness and wellbeing.

**Methods:**

This cross-sectional observational study enrolled participants who were already registered for the Inner Engineering program to learn Shambhavi Mahamudra Kriya, a multi-component, 21-min meditation. Of the 356 participants who consented, 290 participants downloaded the mobile 3Cs app to participate. The enrolled participants were asked to complete self-reported electronic questionnaires at three timepoints: before the program, post-program, and 6 weeks after the program. The following measures were used: the Positive-Negative Relationship Quality (PN-RQ) scale, Interpersonal Mindfulness Scale (IMS), Perceived Stress Scale (PSS), Compassion Scale (CS), and Flourishing Measure. Linear mixed-effects models were used to analyze the survey data, with *p*-values of < 0.05 considered statistically significant.

**Results:**

From the baseline to the 6-week follow-up after the program, the participants reported experiencing more positive qualities (*p* < 0.001, $\eta_{p}^{2}$ = 0.12) and fewer negative qualities (*p* = 0.001, $\eta_{p}^{2}$ = 0.08) in their relationships, increased interpersonal mindfulness (*p* < 0.001, $\eta_{p}^{2}$ = 0.30), decreased stress levels (*p* < 0.001, $\eta_{p}^{2}$ = 0.27), enhanced compassion for others (*p* < 0.001, $\eta_{p}^{2}$ = 0.17), and overall personal wellbeing (*p* < 0.001, $\eta_{p}^{2}$ = 0.25).

**Conclusion:**

Participation in the Inner Engineering yoga program appears to be associated with improvements in relationships, interpersonal mindfulness, compassion, stress, and overall wellbeing.

**Clinical trial registration:**

ClinicalTrials.gov, identifier: NCT05528978.

## 1 Introduction

Interpersonal relationships are a cornerstone of human wellbeing, influencing mental and physical health, success, and overall happiness (Holt-Lunstad et al., 2010; Vaillant, 2012). Acknowledging humanity's inherent social nature, Baumeister and Leary (1995) underscored the importance of positive social bonds for individual thriving (Baumeister and Leary, 1995). Loneliness has become an epidemic, affecting one in three adults (Jeste et al., 2020; National Academies of Sciences, Engineering, and Medicine et al., 2020). This epidemic, along with increased social isolation and dysfunctional social systems, has serious implications for individual and public health (Holt-Lunstad et al., 2010). In fact, a meta-analytic review revealed a 50% increase in survival chances for participants who reported stronger interpersonal relationships, supporting accumulated evidence from epidemiological research that highlights how social isolation and loneliness can increase the risk of morbidity and mortality (Glaser and Kiecolt-Glaser, 1994; Holt-Lunstad et al., 2010).

It is not merely being in relationships that enhances one's health; the quality of the relationship also plays a crucial role in influencing overall wellbeing outcomes. Higher-quality relationships can improve wellbeing, self-esteem (Voss et al., 1999), and life satisfaction (Pateraki and Roussi, 2013; Shek, 1995; Dush and Amato, 2005), whereas poor relationship quality is associated with negative health outcomes, such as an increased risk of premature mortality and higher levels of depressive symptoms (Randall and Bodenmann, 2017; Roberson et al., 2018; South and Krueger, 2013). Perceived stress and relationship quality are also bidirectionally related. Dyadic coping models highlight that perceived stress and the regulation capacity in close relationships mutually influence each other (Bodenmann et al., 2017). The systemic transactional model builds on this by emphasizing the interdependence and reciprocity between partners, where one partner's perceived stress, coping behaviors, and overall wellbeing significantly affect the other partner in a mutually influential manner (Bodenmann et al., 2017). For example, in couples managing illness, increased unsupportiveness from male partners of breast cancer patients is linked to greater illness intrusiveness for the patient (Feldman and Broussard, 2006). As stress is unavoidable in relationships, identifying coping strategies to reduce its impact on individuals and the relationship is important (Randall and Bodenmann, 2017).

Mindfulness practices can enhance both individual wellbeing and relationship quality by fostering present-moment awareness, emotion regulation, and empathy, all of which are qualities that can strengthen interpersonal connections (Barnes et al., 2007). Research shows that mindfulness practices can improve relationship satisfaction, reduce perceived stress, and increase acceptance of others, making them highly relevant for addressing the bidirectional relationship between stress and relationship quality (Barnes et al., 2007; Carson et al., 2004). Notably, even when only one partner engages in a mindfulness intervention, it positively impacts the relationship satisfaction of the non-participating partner (Khaddouma et al., 2017). The effects of mindfulness on stress and relationships extend beyond behavior, leading to measurable changes at the neurological level. For instance, in parent–child relationships, fMRI imaging of parents undergoing the Mindful Families Stress Reduction (MFSR) program showed decreased perceived stress and increased mindfulness, with changes in neural activation in areas linked to empathy and emotion processing/regulation, specifically the left anterior insula/inferior frontal gyrus (May et al., 2016). These changes corresponded to improvements reported by children in aspects of the parent–child relationship such as conflict, monitoring, and positive family relations (May et al., 2016). Mindfulness offers a valuable approach to cultivating the self-regulation and mutual understanding that underpin healthy relationships.

Interpersonal mindfulness is a concept quantified by Pratscher et al. (2018). It applies the qualities of mindfulness to relationships. It is defined as maintaining an open awareness of the ongoing dynamics in interpersonal interactions while being aware of one's own internal experience, practicing non-judgmental acceptance of the other person, responding rather than reacting impulsively, and being sensitive to the changing emotions and needs of others. Although interpersonal mindfulness reflects mindfulness capacity in relationships, studies on how various contemplative practices influence it and relational wellbeing remain limited. One qualitative study of yoga practitioners found that participants reported improved relationships, attributing these improvements to increased patience, kindness, mindfulness, and self-awareness (Ross et al., 2014). Contemplative practices, such as yoga and mindfulness-based stress reduction, may have a profound impact on relational wellbeing and interpersonal health, particularly given that many of these programs focus on improving attentional and regulation capacities, helping people non-judgmentally attend and respond to the present moment.

In this context, Inner Engineering (IE) is a comprehensive program designed to encourage personal growth by exploring the fundamental principles of classical yoga, engaging in meditative practices, and gaining insight into ancient yogic wisdom. The program was developed by Jagadish Vasudev, also known as Sadhguru—a yogi, mystic, and the founder of the Isha Foundation. The foundation is a non-profit, non-religious organization that has been offering tools for wellbeing based on yogic science for over 40 years. Unlike traditional mindfulness programs, which often focus primarily on meditation or cognitive techniques, IE combines multiple dimensions of personal development—cognitive, emotional, and physical components—making it particularly suited for improving individual wellbeing and interpersonal relationships.

The IE program is a self-directed, multi-faceted, secular, and digitally guided program consisting of seven online lessons, each 90 min long, and a live session with Sadhguru to learn the 21-min practice called Shambhavi Mahamudra Kriya (SMK). Following each lesson, participants are encouraged to engage in reflective writing and respond to awareness questions, which allows them to ponder the knowledge gained, apply it to real-life scenarios, and enhance their overall mindfulness. SMK is a practice that consists of alternate nostril breathing, aum chanting, and breath watching. This is currently being offered as Seven Steps with Sadhguru, and all programs, including the 21-min practice, can be done online.

The program is delivered through the Isha Foundation and includes four key components: (1) reevaluating cognitive perspectives and core beliefs, (2) fostering positive emotions through Hatha Yoga, which incorporates body movements and breath techniques, (3) facilitating guided meditation, and (4) stimulating inner energy through a combination of sound and yoga, with the aim of evoking emotional empathy, body relaxation, and mental attentiveness (Upadhyay et al., 2022a,b). These elements collectively aim to equip individuals with the tools to enhance their regulatory capacity and navigate relationships with greater emotional clarity and resilience.

Studies on IE have shown that it can enhance overall wellbeing and mindfulness and reduce stress (Upadhyay et al., 2022a,b). However, limited studies have explored the impact of SMK on relationships. One study examined the short-term impact of SMK on IE program participants over a 6-week period and found a significant increase in relationship satisfaction. However, the results at these timepoints showed only slight improvements over the short study duration and were based on a small sample size (Upadhyay et al., 2022b).

To understand the impact of IE on interpersonal functioning, the present study explored the potential effects of IE on interpersonal relationships and interpersonal mindfulness. Specifically, we hypothesized that participants in IE would report improvements in relationship quality, interpersonal mindfulness, compassion for others, overall wellbeing, and perceived stress. This study sought to fill a critical gap in the limited research on IE's influence on relationship dynamics, with potential implications for addressing broader social challenges such as loneliness and the emotional wellbeing of individuals in relational contexts. This research aimed to demonstrate IE's ability to improve relationship quality, providing a foundation for future studies to explore its potential as a tool for enhancing interpersonal connections and fostering healthier, more resilient social environments.

## 2 Methods

### 2.1 Participants and procedure

This cross-sectional observational study enrolled participants who were already registered for the Inner Engineering program to learn SMK and were proficient in English. Participants were excluded from the study if they were not currently residing in the United States and were under the age of 18.

In this study, 356 participants met the eligibility criteria and consented to participate. Participants who did not download and enroll in the 3Cs app, complete the baseline survey, and respond to the follow-up survey confirming their completion of the IE program, as well as those who explicitly stated they did not complete the program, were excluded from the study. We had a final sample size of 290 participants. The sample size per timepoint varied due to incomplete participant responses or participant inactivity. See Table 1 for the sample size breakdown per survey and timepoint.

**Table 1:** Median and IQR of the surveys.

Scales (*N*) ^*^	**Baseline**	**Post-program**	**Week 6**	***p*-value^a^**	**Effect size^b^**
PN-RQ (*N*)	179	171	129		
Positive	21 (16, 31)	25 (18, 33)	28 (21, 36)	< 0.001	0.12
Negative	6 (2, 13)	4 (1, 12)	2 (0, 7)	< 0.001	0.08
IMS (*N*)	190	177	133		
Total	92 (84, 105)	103 (90, 113)	110 (96, 120)	< 0.001	0.30
Presence	22 (19, 25)	25 (21, 28)	26 (23, 30)	< 0.001	0.30
Awareness of self and others	38 (33, 43)	40 (35, 45)	42 (37, 47)	< 0.001	0.19
Non-judgmental acceptance	14 (12, 16)	15 (13, 17)	16 (14, 18)	< 0.001	0.17
Non-reactivity	21 (18, 23)	22 (19, 25)	24 (21, 27)	< 0.001	0.19
PSS (*N*)	173	167	127		
Total	20 (15, 25)	16 (11, 21)	12 (8, 17)	< 0.001	0.27
Compassion scale (*N*)	160	164	123		
Total	63 (58, 69)	65 (59, 70)	69 (63, 74)	< 0.001	0.17
Indifference	15 (13, 17)	16 (14, 17)	16 (14, 18.5)	0.003	0.05
Kindness	16 (14, 19)	17 (15, 19)	18 (16, 20)	< 0.001	0.12
Mindfulness	16 (14, 18)	17 (15, 18.75)	18 (16, 20)	< 0.001	0.22
Common humanity	16 (14, 18)	16 (15, 18)	18 (15, 19.5)	0.021	0.03
Flourishing measure (*N*)	215	186	141		
Happiness and life satisfaction	13 (11, 15)	15 (12, 17)	16 (14, 18)	< 0.001	0.16
Mental and physical health	13 (11, 16)	14 (12, 17)	16 (14, 18)	< 0.001	0.20
Meaning and purpose	13 (10, 15)	14 (12, 17)	16 (14, 18)	< 0.001	0.25
Character and virtue	14 (11, 16)	15 (13, 17)	17 (14, 18)	< 0.001	0.11
Close social relationships	12 (9, 14)	14 (10, 16)	15 (13, 18)	< 0.001	0.11
Financial and material stability	15 (11, 18)	16 (12, 18)	18 (15, 19)	< 0.001	0.06

^a^p-value from the linear mixed-effects model (Time baseline vs. Week 6).

^b^Partial eta squared.

^*^N value varies at each timepoint due to participant activity variability.

The researchers worked with the Isha Foundation to recruit program participants through flyers, emails, and booths. To participate, individuals were required to provide electronic prospective consent through REDCap. They were then asked to download the 3Cs app on their smartphone to complete the study surveys. The 3Cs app is a mobile app designed for researchers to conduct studies and allows participants to respond to surveys on their smartphones. The app is approved by BIDMC information systems for research data collection purposes. It collects data from participants and stores it in a HIPAA-compliant database called Google Firebase. During the study, a few participants experienced minor technical issues during the enrollment and while submitting the surveys via the app. These issues were promptly addressed and resolved by the app support team.

**Figure 1:**
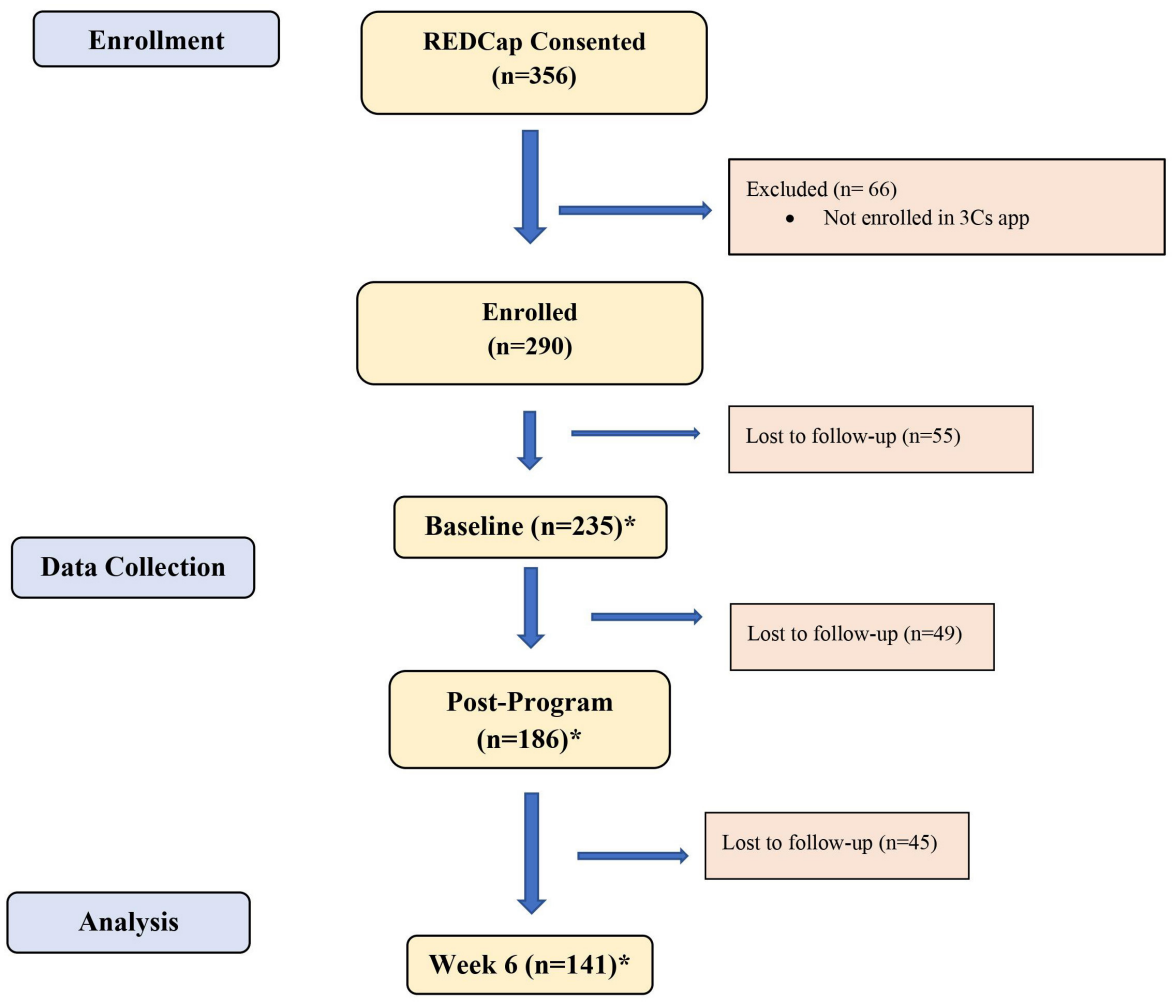
CONSORT flow diagram. ^*^The total number reported at each timepoint reflects the largest *N* for the survey completed at that timepoint. There is variation in the number of the participants completing each survey at each timepoint due to varying completion rates and participant drop-off.

The study surveys were provided at Baseline (T1), within a week after the program (T2), and 6 weeks after the program (T3). The surveys included the Positive and Negative Relationship Quality (PN-RQ) scale, Interpersonal Mindfulness Scale (IMS), Perceived Stress Scale (PSS), Flourishing Measure, and Compassion Scale (CS). Self-reported weekly activity logs, tracking the participants' practice frequency, were provided for 6 weeks (see Figure 1).

### 2.2 Measures

#### 2.2.1 Relationship quality

Relationship quality was assessed using the PN-RQ scale (Rogge et al., 2017). The PN-RQ scale measures perceptions of relationship quality, with eight items addressing positive aspects (e.g., “enjoyable” and “energizing”) and eight items focusing on negative aspects (e.g., “empty” and “discouraging”). The participants provided responses on a scale ranging from 0 (Not at all true) to 5 (Completely true). The total scores for each aspect were summed. Higher scores on the positive subscale indicated increased positive relationship qualities, while higher scores on the negative subscale denoted heightened negative relationship qualities. The reliability of the PN-RQ positive subscale was 0.94 at the baseline, 0.96 post-program, and 0.97 at week six. The reliability of the PN-RQ negative subscale was 0.94 at the baseline, 0.95 post-program, and 0.96 at week six (see Supplementary Table S1).

#### 2.2.2 Interpersonal mindfulness

The IMS was employed to assess mindfulness in interpersonal interactions (Pratscher et al., 2019). This scale evaluates four aspects of mindfulness: attention to the present moment, awareness of self and others, non-judgmental acceptance, and non-reactivity. A validation study conducted by Medvedev et al. (2020), utilizing Rasch analysis to investigate psychometric properties, confirmed the IMS's robust reliability and internal validity. Previous research, controlling for mindfulness as a trait, has linked interpersonal mindfulness to friendship quality outcomes (Pratscher et al., 2018). The scale comprises 27 items, each rated on a 5-point Likert scale ranging from 1 (almost always) to 5 (almost never). The sample items include “Before I speak, I am aware of the intentions behind what I am trying to say” and “When I am upset with someone, I notice how I am feeling before responding.” The reliability of the IMS was 0.95 at the baseline; 0.95 post-program, and 0.96 at week 6 (see Supplementary Table S1).

#### 2.2.3 Perceived stress

Stress levels were evaluated using the PSS (Cohen et al., 1983). This scale assesses the frequency of experiencing specific stressors or thinking about stressful events in the past month. Widely utilized in stress-related research, the PSS has well-established reliability and validity. This 10-item scale employs a 5-point Likert scale, ranging from 0 (never) to 4 (very often). All responses are then aggregated to derive a stress score within a range of 0 to 40. One of the sample items is “In the last month, how often have you been upset because of something that happened unexpectedly?” The reliability of the PSS was 0.92 at the baseline; 0.93 post-program, and 0.90 at week six (see Supplementary Table S1).

#### 2.2.4. Compassion for others

Compassion levels were evaluated using the Compassion Scale (CS) (Pommier et al., 2020). This scale measures compassion for others and draws on Neff's conceptual framework of self-compassion. This 16-item scale uses a 5-point Likert scale, ranging from 1 (almost never) to 5 (almost always). All responses were then aggregated to derive a mean score. One of the sample items is “I realize everyone feels down sometimes, it is part of being human.” The reliability of the CS was 0.80 at the baseline; 0.86 post-program, and 0.87 at week 6 (see Supplementary Table S1).

#### 2.2.5 Overall wellbeing

General wellbeing was measured using the Flourishing Measure (VanderWeele, 2017). This scale examines wellbeing across the following six domains: happiness and life satisfaction, mental and physical health, meaning and purpose, character and virtue, close social relationships, and financial and material stability. Along with a total mean score, each domain is also evaluated separately. Each domain consists of two questions, scored on a scale from 0 to 10. For example, a question in the happiness and life satisfaction domain is “Overall, how satisfied are you with life as a whole these days?” (0 = not satisfied at all, 10 = completely satisfied). The reliability of the Flourishing Measure was 0.80 at the baseline; 0.82 post-program, and 0.86 at week 6 (see Supplementary Table S1).

### 2.3 Data analyses

Repeated measure analyses were performed using linear mixed-effects models, adjusting for age, sex, ethnicity, education, and previous yoga experience as fixed effects, the interaction term of time (baseline, post-session, and week 6), study compliance indicator, and random intercepts shared by the individuals as random effects. The interaction term was used to assess the differences in the outcome changes between the compliant and non-compliant participants across the timepoints. Partial eta squared were computed as an effect size, with 0.02– < 0.13 considered a small effect, 0.13– < 0.26 a medium effect, and ≥0.26 a large effect. Models including the interaction term of compliance and time were also considered to assess whether the outcome changes differed by compliance status. All statistical analyses were performed using R (version 4.3.2, The R Foundation for Statistical Computing), and two-sided *p*-values of 0.05 were considered statistically significant for all tests except for the interaction terms (*p* < 0.1). Continuous data were presented as medians and interquartile ranges (IQR). Categorical data were presented as frequencies and proportions (%).

## 3 Results

### 3.1 Demographics

Based on the overall sample size (*n* = 235) of the participants who completed the surveys at each timepoint, the sample was primarily female (60%), Asian (60%), and of non-Hispanic ethnicity (85%). A significant portion had a graduate degree (43%) and worked full-time (55%). The majority of the participants were married or in a domestic partnership (57%) and had one or more children (53%; Table 2, Supplementary Table S2 for previous yoga experience).

**Table 2:** Sociodemographic characteristics of the participants at the baseline.

**Baseline characteristics**	***n***	**%**
**Sex**		
Female	142	60
Male	92	39
Other	1	0.4
**Race**		
Asian	140	60
Black or African American	8	3.4
Multi-Racial	14	6.0
Native Hawaiian or Other Pacific Islander	1	0.4
Other	12	5.1
Prefer not to specify	3	1.3
White	57	24
**Ethnicity**		
Hispanic or Latino	23	9.8
Not Hispanic or Latino	200	85
Prefer not to specify	12	5.1
**Education**		
Associate	12	5.1
Doctoral	29	12
Graduate	100	43
High school/GED	20	8.5
Prefer not to specify	2	0.9
Trade school	7	3
Undergraduate	65	28
**Employment**		
Contingent worker	4	1.7
Disabled, not able to work	2	0.9
Full-time	130	55
Laid off	2	0.9
Military service	1	0.4
Not employed, looking for work	11	4.7
Not employed, not looking for work	2	0.9
Other	2	0.9
Part-time	21	8.9
Retired	5	2.1
Self-employed	48	20
Student	7	3
**Working hours**		
>40 h/week	103	44
0–20 h/week	35	15
20–40 h/week	97	41
**Marital status**		
Divorced	31	13
Married or domestic partnership	134	57
Separated	6	2.6
Single, never married	63	27
Widowed	1	0.4
**Number of children**		
1	37	16
2–4	85	36
More than 4	2	0.9
None	111	47

N = 235 (n = 39 for each condition). The participants' median age was 39 years old (IQR = 33, 48).

### 3.2 Relationship quality

**Figure 2:**
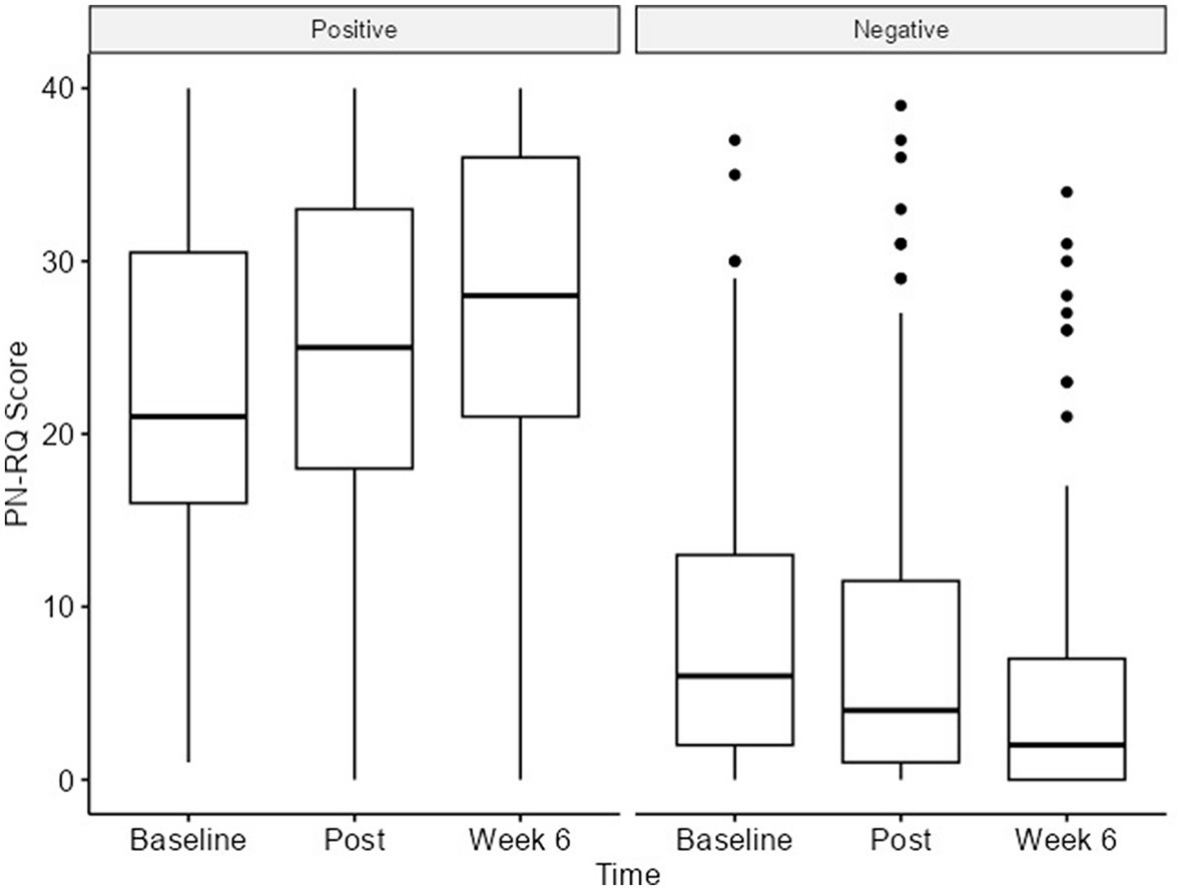
PN-RQ (positive and negative) scores at all timepoints.

Analyses for the PN-RQ scale were performed using repeated measures analysis with a linear mixed-effects model. We observed statistically significant differences in both the positive and negative components, with increasing/reducing scores showing small effect sizes. The PN-RQ positive subscale changed from the baseline [21 (IQR 16–31)] to the 6-week follow-up [28 (IQR 21–36)], reflecting a 7-point increase with a *p*-value < 0.001. The PN-RQ negative subscale changed from the baseline [6 (IQR 2–13)] to the 6-week follow-up [2 (IQR 0–7)], reflecting a 4-point decrease with a *p*-value < 0.001 (Table 1, Figure 2, Supplementary Table S4; Supplementary Figures S1, S2).

### 3.3 Interpersonal mindfulness

**Figure 3:**
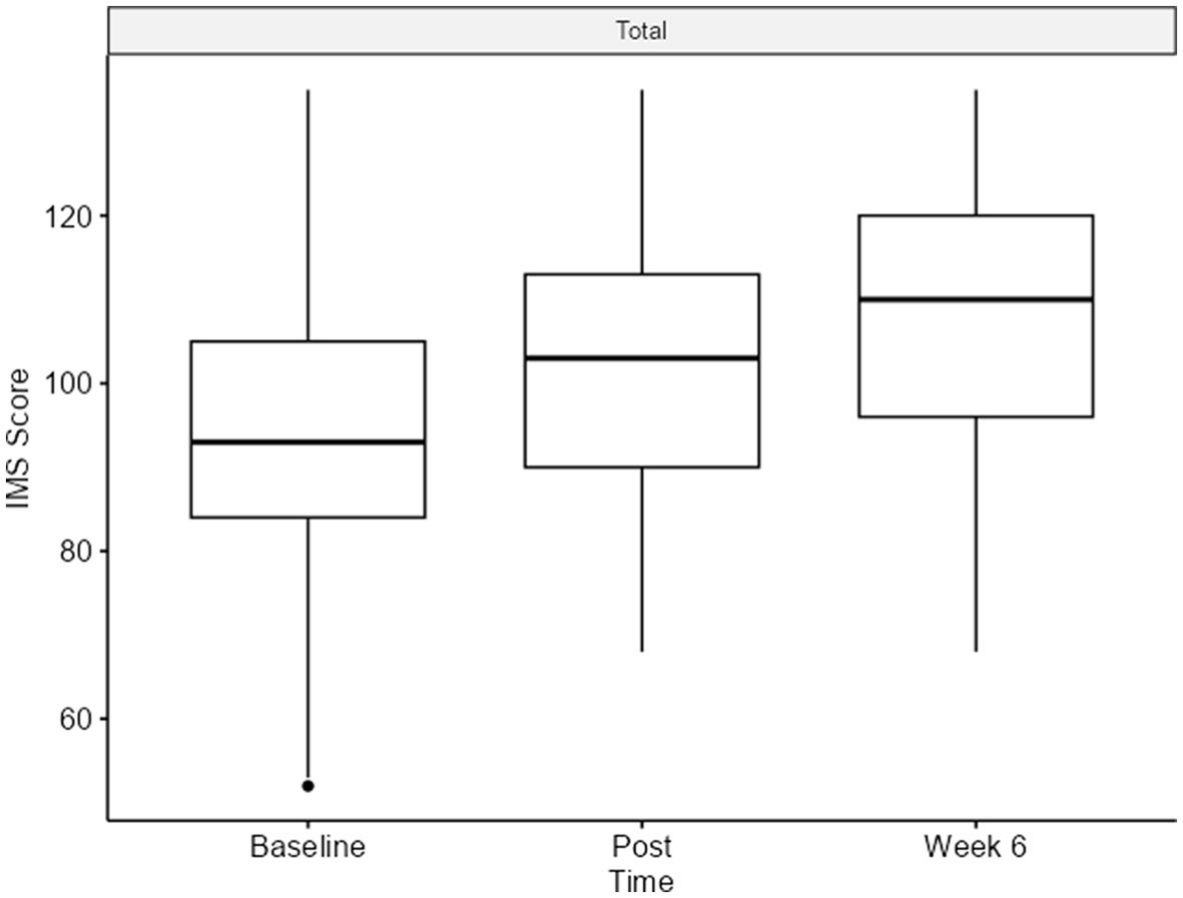
Interpersonal mindfulness scale (total) at all timepoints.

The IMS scale was analyzed using a linear mixed-effects model. We observed statistically significant differences in the total score, with an increase in direction and a large effect size. The change from the baseline [92 (IQR 84–105)] to post-program [103 (IQR 90–113)] was an 11-point increase. From post-program [103 (IQR 90–113)] to the 6-week follow-up [110 (IQR 96–120)], a 7-point increase was observed with a *p*-value < 0.001 (Figure 3).

The presence subscale ([22 (19–26)] to [26 (23–30)]; *p* < 0.001; $\eta_{p}^{2}$ = 0.28) showed significant differences from the baseline to the 6-week follow-up with a large effect size. The following subscales showed significant differences from the baseline to the 6-week follow-up with medium effect size changes: awareness of self and others ([38 (33–43)] to [42 (37–47)]; *p* < 0.001; $\eta_{p}^{2}$ = 0.19), non-judgmental acceptance ([14 (12–16)] to [16 (14–18)]; *p* < 0.001; $\eta_{p}^{2}$ = 0.17), and non-reactivity ([21 (18–23)] to [24 (21–27)]; *p* < 0.001; $\eta_{p}^{2}$ = 0.19; Table 1 and Figure 3; Supplementary Figure S6).

### 3.4 Perceived stress

**Figure 4:**
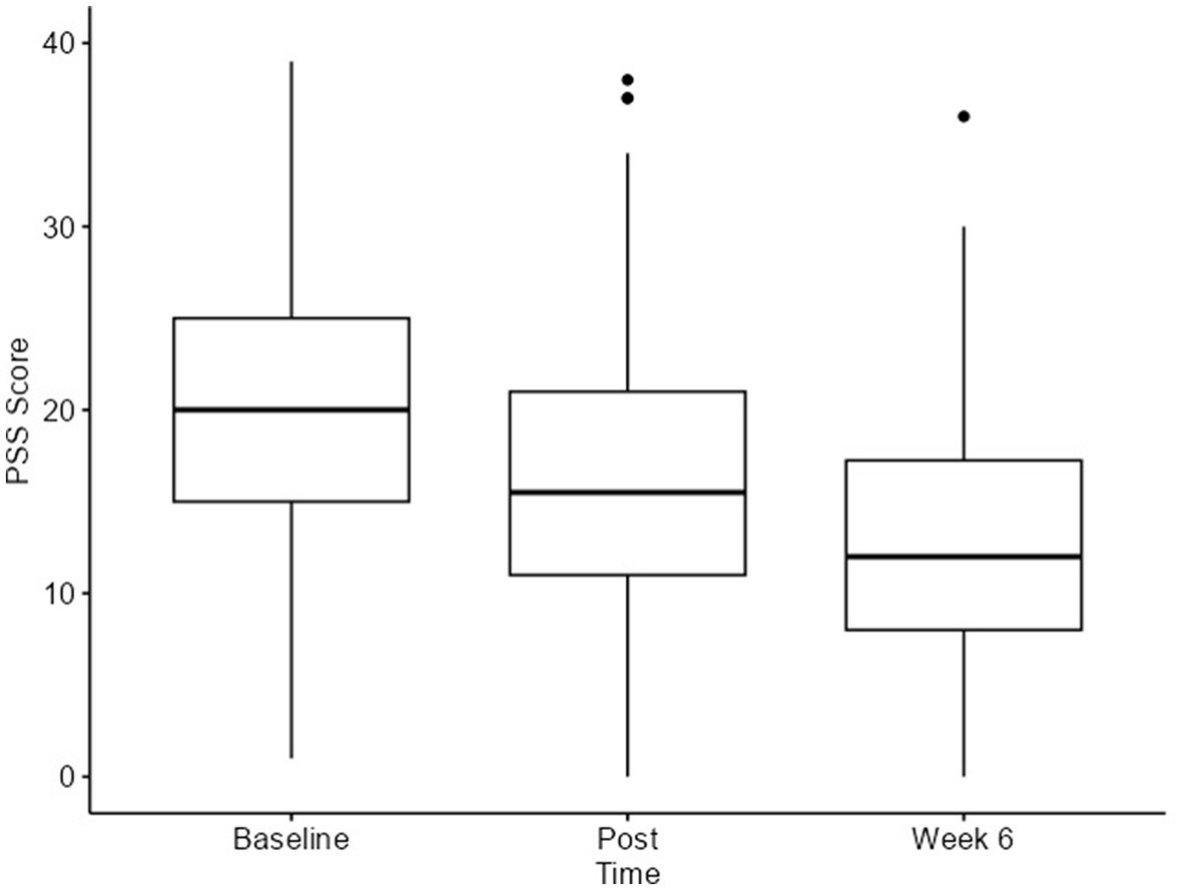
PSS score at all timepoints.

The PSS scale was analyzed using a linear mixed-effects model. We observed statistically significant differences in the PSS scores, with a reduction in the stress scores and a large effect size between the baseline and the six-week follow-up. The change from the baseline [20 (IQR 15–25)] to post-program [16 (IQR 11–21)] was a 4-point decrease. From post-program [16 (IQR 11–21)] to the 6-week follow-up [12 (IQR 8–17)], a 4-point decrease was observed with a *p*-value < 0.001 (Table 1 and Figure 4).

### 3.5 Compassion for others

**Figure 5:**
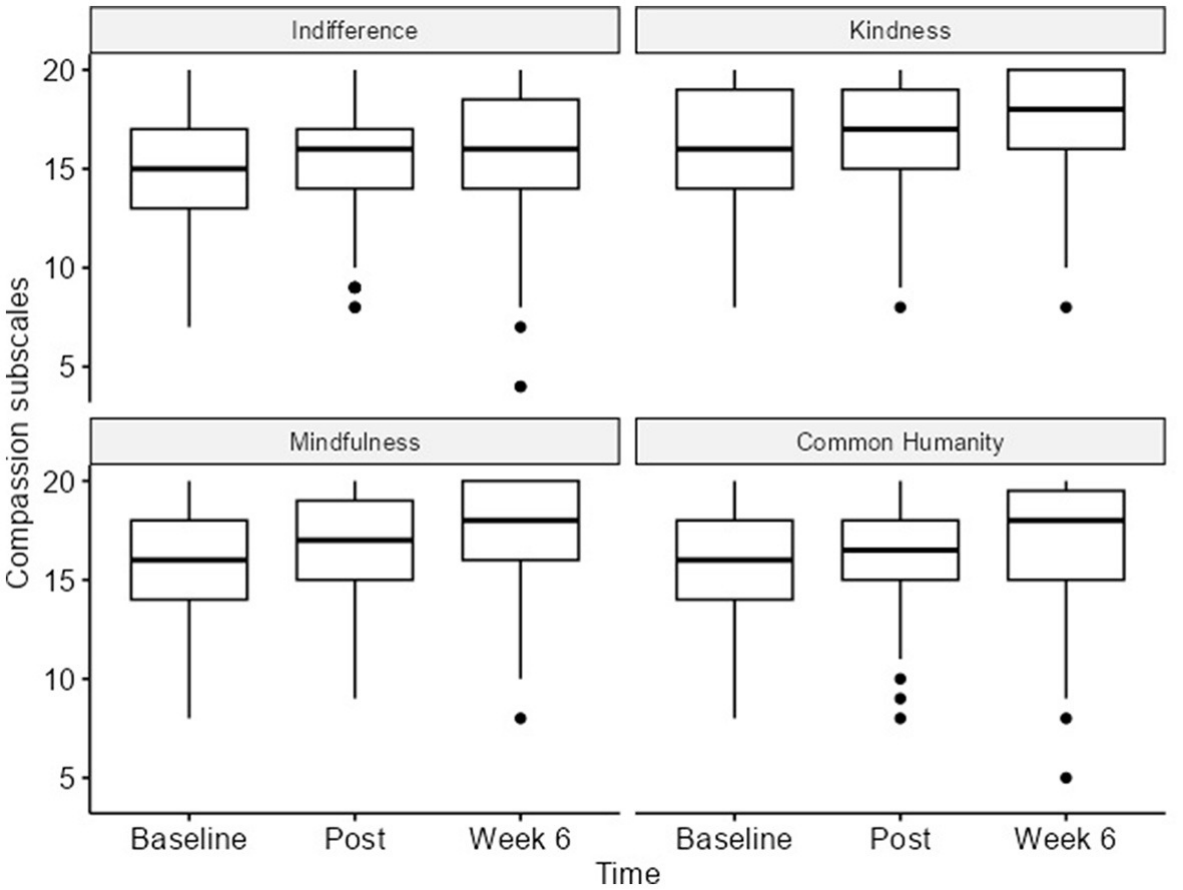
Compassion scale (total) at all timepoints.

The CS was analyzed using a linear mixed-effects model. We observed statistically significant increases in the total compassion score, with an increase in direction and a medium effect size between the baseline and six-week follow-up. The change from the baseline [63 (IQR 58–69)] to post-program [65 (IQR 59–70)] was a 2-point increase. From post-program [65 (IQR 59–70)] to the 6-week follow-up [69 (IQR 63–74)], a 4-point increase was observed with a *p*-value < 0.001 (Figure 5).

The compassion survey subscales showed statistically significant differences from the baseline to the 6-week follow-up, with a medium effect size for mindfulness ([16 (14–18)] to [18 (16–20)]; *p* < 0.001; $\eta_{p}^{2}$ = 0.22) and small effect sizes for indifference ([15 (13–17)] to [16 (14–18.5)]; *p* < 0.001; $\eta_{p}^{2}$ = 0.05), kindness ([16 (14–19)] to [18 (16–20)]; *p* < 0.001; $\eta_{p}^{2}$ = 0.12), and common humanity ([16 (14–18)] to [18 (15–19.5)]; *p* < 0.02; $\eta_{p}^{2}$ = 0.03; Table 2; Supplementary Figure S8).

### 3.6 Overall wellbeing

**Figure 6:**
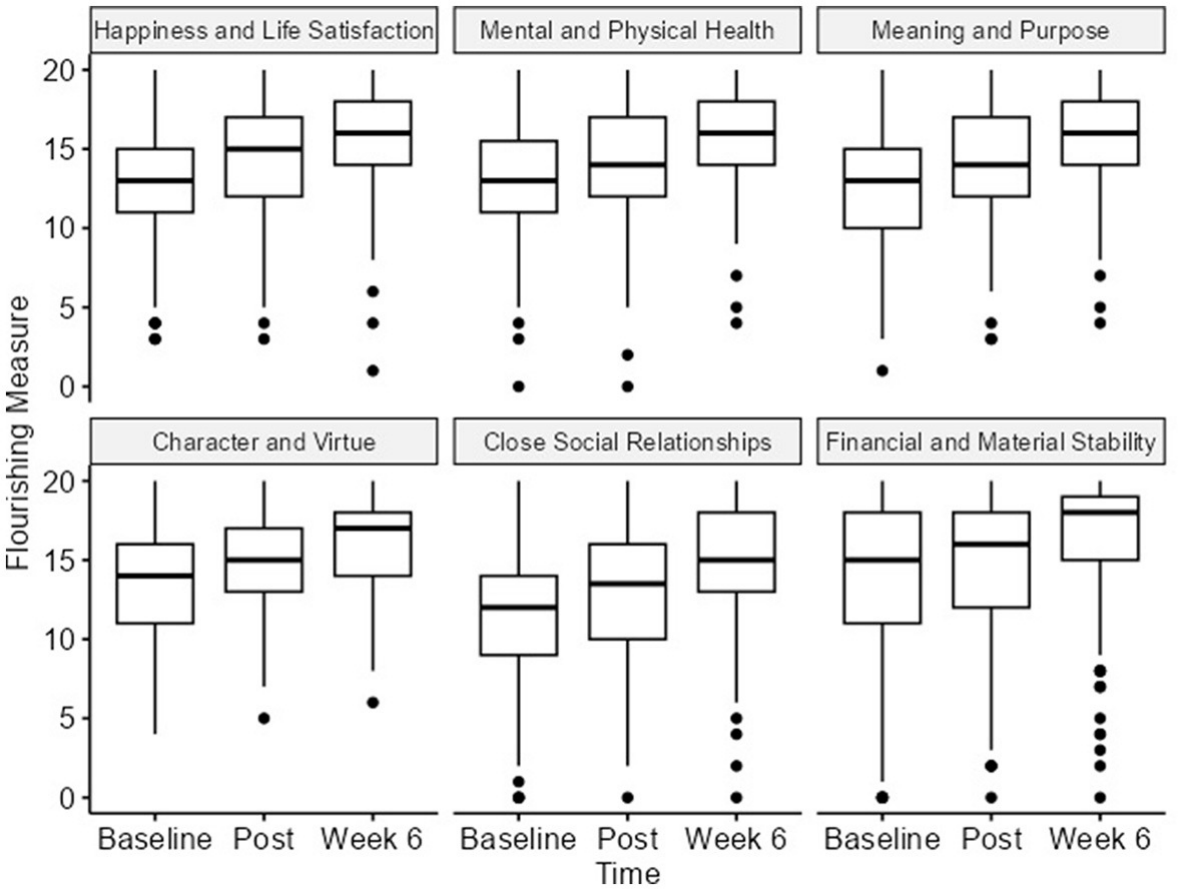
Flourishing measure at all timepoints.

Each component of the Flourishing Measure scale was analyzed using repeated measure analysis with a linear mixed-effects model. We observed statistically significant differences in every domain, with an increase in direction and small to medium effect sizes between the baseline and the 6-week follow-up. The close social relationships domain significantly increased from the baseline [12 (IQR 9–14)] to the 6-week follow-up [15 (IQR 13–18)], with a *p*-value of 0.001 and a small effect size ($\eta_{p}^{2}$ = *0.1*1; Table 1 and Figure 6).

### 3.7 Compliance

Compliance with the practice of SMK at least four times a week for 4 weeks is a key measure for evaluating the efficacy of the practice. Regular practice of SMK is important for deriving deeper benefits. Variations in compliance can influence the extent of the benefits participants experience. A model with the interaction term of compliance status was used. Further analysis showed no significant differences in the demographic and baseline conditions contributing to the different levels of compliance. For relationship quality, the compliant participants experienced a more significant change in the PN-RQ positive scale items at 6 weeks for “fun” (*p* = 0.047) and “exciting” (*p* = 0.026) compared to the non-compliant participants (Supplementary Figures S3–S5). A significant change was also observed in the total IMS score (*p* = 0.058). The compliant participants showed significant changes in awareness of self and others (*p* = 0.017), non-judgmental acceptance (*p* = 0.002), and non-reactivity (*p* = 0.028) throughout the study period, compared to the non-compliant participants (Supplementary Figure S7). No significant change was observed in perceived stress. For compassion, an interaction effect by compliance status was found on indifference (*p* = 0.05). In the Flourishing Measure, the compliant participants showed more significant changes in the following domains: mental and physical health (*p* = 0.064), close social relationships (*p* = 0.027), and financial and material stability (*p* = 0.062; Supplementary Figures S9–S11; See Supplementary Table S4 for all compliance outcomes).

### 3.8 Correlations among outcomes

Correlation analyses among the changes in the outcomes were conducted from the baseline to the 6-week follow-up. The total IMS score changes showed moderate correlations between the changes in the positive PN-RQ (*r* = 0.48, *p* < 0.001), the total PSS score (*r* = −0.58, *p* < 0.001), and the total compassion score (*r* = 0.59, *p* < 0.001). The changes in the positive PN-RQ showed moderate correlations between the changes in the negative PN-RQ (*r* = −0.53, *p* < 0.001), the social relationships domain in the Flourishing Measure (*r* = 0.55, *p* < 0.001), the total PSS Score (*r* = −0.41, *p* < 0.001), and the total compassion score (*r* = 0.50, *p* < 0.001) (Supplementary Table S5).

## 4 Discussion

In the present study, we examined the impact of the Inner Engineering program on interpersonal relationships. We hypothesized that participation in the Inner Engineering program could improve interpersonal wellbeing, such as relationship quality, interpersonal mindfulness, compassion for others, and perceived stress. The results demonstrated increases in various dimensions, including improvements in relationship quality, interpersonal mindfulness (IMS), overall wellbeing, compassion, and perceived stress. As expected, the moderate correlations between the changes in the Positive-Negative Relationship Quality Scale (PN-RQ) and the remaining scales align with existing literature, which shows that relationship quality is positively associated with mindfulness, wellbeing, compassion, and stress reduction (Dush and Amato, 2005; Jiang et al., 2020; Khaddouma et al., 2017; Randall and Bodenmann, 2017).

The improvement in relationship quality, characterized by a significant increase in perceived positive qualities and a decrease in perceived negative qualities, can be contextualized within the broader framework of how mindfulness practices enhance wellbeing and mood, possibly giving way to more compassionate interpretations of interpersonal interactions and stronger interpersonal emotion regulation skills. Furthermore, the improvement in interpersonal mindfulness is supported by previous research demonstrating that the Inner Engineering program enhances intrapersonal mindfulness (Upadhyay et al., 2022b). By cultivating mindfulness through yoga, a chain reaction of positive improvements can occur, extending from one's relationship with oneself to their relationships with others (Kishida et al., 2019). Although this study could not determine if interpersonal mindfulness causes an increase in relationship quality, the two are positively correlated. The literature has expanded on this connection by linking mindfulness to positive relationship outcomes through improved effective communication, mitigation of interpersonal conflicts, enhanced ability to cope with stress, and an increased willingness to engage in authentic relationships (Brunell et al., 2010; Burgoon et al., 2000; Carson et al., 2004; Head and Hammer, 2013).

The breath-based components of SMK may also play a role in increasing interpersonal mindfulness. A study comparing breath-focused mediation and emotion-focused meditation found that the breath-focused group exhibited greater emotional stability, reduced reactivity, and a higher willingness to engage with negative stimuli (Arch and Craske, 2006). This practice can lead to a more adaptive response to uncomfortable emotions or situations in interpersonal relationships (Arch and Craske, 2006). When individuals are able to recognize and regulate distress, they create space for experiencing positive emotions more frequently. It is interesting to note that in the PN-RQ Positive scale, the compliant participants showed more significant changes over time than the non-compliant participants. In the PN-RQ Positive scale, the overall changes were not observed based on compliance status. However, the participants reported experiencing more “fun” and “exciting” qualities in their relationships. This implies that regular practice could contribute to greater changes in relationship dynamics, such as experiencing more positive qualities.

Our results showed significant stress reduction and improved relationship quality among the IE program participants. This finding is supported by the literature, which has established an inverse relationship between stress and relationship quality, as well as a spillover effect, whereby the experience of stress can extend or “spillover” from one life domain to interpersonal relationships (Randall and Bodenmann, 2017). As Randall and Bodenmann (2017) highlighted, understanding coping strategies is pivotal when exploring the interplay between stress and relationship quality. Studies have indicated that mindfulness practices can effectively lower stress levels, enhance coping mechanisms, and elevate relationship satisfaction (Carson et al., 2004). Inner Engineering can be an effective coping strategy for stress, thereby helping enhance interpersonal relationship functioning. Although our study did not directly assess the impact on the partners of the participants, stress is a dyadic phenomenon, where individuals mutually influence each other's experiences (Randall and Bodenmann, 2017). Consequently, enhanced stress coping in one person can positively affect the relationship as a whole (Randall and Bodenmann, 2017). Future research should more specifically seek to assess the experiences of individuals in interpersonal relationships undergoing the Inner Engineering program to deepen our understanding of the interplay between stress and relationship quality within this practice.

Compassion levels significantly increased between the post-program assessment and the 6-week follow-up. Increased compassion was also found to be positively correlated with relationship-associated positive qualities and interpersonal mindfulness. The conceptual definition of compassion entails qualities that can increase the likelihood of positive dyadic coping styles in relationships, such as common humanity, kindness, and mindfulness. Increased common humanity and kindness foster greater empathy for the other person's challenges and reflect a desire to offer a supportive behavioral response. Furthermore, mindfulness involves being present in the moment, practicing non-reactivity, and adopting a non-judgmental stance toward the other person's tendencies, beliefs, habits, and moods. This can support more effective emotion regulation, thereby reducing the likelihood of feeling overwhelmed by the other person's stressors. It can also lead to more supportive communication styles by encouraging attentiveness to others, listening without criticism or judgment, being aware of one's own needs and emotions, as well as those of others, and refraining from impulsive reactions. In marital relationships, compassion has been shown to enhance relationship quality by promoting supportive dyadic coping styles (Collins, 2014; Jiang et al., 2020). Individuals felt more empathy for their partner going through a stressful life event, which, in turn, predicted increased affection and care for them (Collins, 2014).

### 4.1 Strengths, limitations, and future directions

The strength of this study lies in its ability to link multiple interpersonal measures, such as interpersonal mindfulness and relationship quality, to the Inner Engineering program, helping pave the way for future studies on mindfulness and interpersonal functioning. Moreover, by using a mobile app and administering this program online, we provided the participants with a more convenient method for responding to the surveys and engaging in the research. Unfortunately, the lack of a control group is a limitation, making it difficult to determine whether the impact of IE on our outcomes was causal or simply correlational and due to extraneous variables. Furthermore, the differences observed between the baseline and post-program timepoints, along with similar outcomes based on compliance for certain scales, suggest that further research is needed to understand the distinct influence of SMK on participants compared to those who only experienced the Inner Engineering online program.

In addition, in the compassion survey, the compassion measure used in the study does not capture the translation of compassionate feelings into observable behaviors. To address this limitation, future research should consider employing more comprehensive measures that effectively capture the behavioral dimensions of compassion, thus providing a more nuanced understanding of its manifestation and development within the context of programs such as Inner Engineering.

In addition, the current study relied on self-reported measures from the participants, lacking input from individuals within their social circles, which could have introduced biased outcomes. Statistical analysis using repeated measures in the same individuals over a 6-week time frame was intended to improve data analysis rigor of the self-reported data and mitigate the lack of randomization and a control group. Future studies on interpersonal relationships with IE practitioners should include individuals from their social networks to more comprehensively examine SMK's impact on both practitioners and non-practitioners and its influence on relationship quality.

Overall, this study contributes to current research. It shows that mind-body practices, such as Inner Engineering /Shambhavi Mahamudra Kriya, may significantly enhance relationship quality, deepen interpersonal mindfulness, nurture interpersonal compassion, and reduce stress within 6 weeks. It also highlights the practical potential of Inner Engineering to enhance relationship quality, offering valuable insights for both practitioners and individuals.

## Data Availability

The raw data supporting the conclusions of this article will be made available by the authors, without undue reservation.
