# Supplementary material for: Inner engineering is associated with perceived improvements in relationship quality, interpersonal connections, and interpersonal compassion
Source: Front Psychol. 2024 Dec 23;15:1454293. doi: 10.3389/fpsyg.2024.1454293 (PMC11701375; doi:10.3389/fpsyg.2024.1454293)

**Supplementary Material for Inner Engineering and Interpersonal Relationships Manuscript**

**Supplementary Table 1**

Internal Consistency using Cronbach's alpha for outcome measures

|  | Baseline | Post session | Week 6 |
| --- | --- | --- | --- |
| PSS | .92 | .93 | .90 |
| PN-RQ Positive | .94 | .96 | .97 |
| PN-RQ Negative | .94 | .95 | .96 |
| IMS |  |  |  |
| Total | .95 | .95 | .96 |
| Presence | .89 | .88 | .91 |
| Awareness of Self Awareness | .91 | .92 | .93 |
| Non-judgmental acceptance | .78 | .82 | .85 |
| Non-reactivity | .84 | .86 | .87 |
| Compassion scale |  |  |  |
| Total | .80 | .86 | .87 |
| Indifference | .69 | .75 | .77 |
| Kindness | .81 | .79 | .83 |
| Mindfulness | .79 | .78 | .76 |
| Common humanity | .71 | .61 | .77 |
| Flourishing Measure | .80 | .82 | .86 |

**Supplementary Table 2**

Previous Yoga Experience at Baseline

|  | n | % |
| --- | --- | --- |
| Previous Yoga experience | 129 | 75 |
| Yoga Schools |  |  |
| Isha Foundation | 45 | 26 |
| Brahma Kumari | 6 | 3.5 |
| Art of living | 13 | 7.6 |
| Zen meditation | 16 | 9.4 |
| Mindfulness Based Stress Reduction | 24 | 14 |
| Other | 83 | 49 |
| Isha Foundation Yoga practice past month |  |  |
| Aum Chanting | 34 | 20 |
| Upa Yoga | 11 | 6.4 |
| Isha Kriya | 24 | 14 |
| Simha Kriya | 0 | 0 |
| Other | 11 | 6.4 |
| Isha Foundation Yoga practice frequency past month |  |  |
| 1 ≤ days in a week | 13 | 29 |
| 2-3 days in a week | 10 | 22 |
| 4-5 days in a week | 11 | 24 |
| ≥ 6 days in a week | 11 | 24 |

*Note.* Participants had a median of 3 years of yoga experience (IQR=0, 5). Participants practiced a median of 2 hours of yoga per week (IQR=0, 5).

**Supplementary Table 3**

*Outcomes by Compliance*

|  | Compliant | | | Not complaint | | | p-value | |
| --- | --- | --- | --- | --- | --- | --- | --- | --- |
| Scales (N) * | Baseline | Post | Week 6 | Baseline | Post | Week 6 | Post | Week 6 |
| Flourish Measure (N) | 78 | 102 | 95 | 74 | 68 | 46 |  |  |
| Happiness & Life Satisfaction | 14 (12, 15) | 15 (13, 17) | 16 (14, 17) | 13 (10, 16) | 15 (12, 17) | 16 (13, 18) | .8 | .4 |
| Mental & Physical Health | 13 (11, 16) | 15 (12, 17) | 16 (14, 18) | 13 (11, 16) | 14 (12, 16) | 16 (13, 18) | .6 | .064 |
| Meaning & Purpose | 13 (10, 15) | 14 (12, 17) | 16 (14, 18) | 13 (10, 16) | 14 (12, 17) | 16 (13, 18) | .5 | .4 |
| Character & Virtue | 15 (12, 16) | 15 (13, 17) | 17 (15, 18) | 14 (10, 16) | 15 (12, 17) | 17 (14, 18) | .5 | .7 |
| Close Social Relationships | 12 (10, 16) | 14 (10, 16) | 16 (14, 18) | 12 (9, 14) | 13 (10, 17) | 14 (11, 18) | .3 | .027 |
| Financial & Material Stability | 15 (11, 18) | 16 (12, 18) | 18 (16, 20) | 15 (12, 18) | 15 (12, 18) | 17 (11, 18) | .3 | .062 |
| PSS (N) | 73 | 102 | 90 | 62 | 63 | 37 |  |  |
| Total | 20 (13, 24) | 17 (11, 22) | 12 (8, 17) | 19 (15, 25) | 14 (11, 20) | 13 (9, 17) | .3 | .3 |
| PN-RQ (N) | 74 | 102 | 92 | 65 | 67 | 37 |  |  |
| Positive | 21 (16, 31) | 25 (20, 32) | 30 (23, 36) | 22 (15, 32) | 27 (16, 36) | 24 (16, 35) | .13 | .2 |
| Enjoyable | 3 (2, 4) | 3 (3, 4) | 4 (3, 5) | 3 (2, 4) | 4 (2, 5) | 3 (2, 4) | .2 | .5 |
| Pleasant | 3 (2, 4) | 3 (3, 4) | 4 (3, 5) | 3 (2, 5) | 4 (2, 5) | 3 (3, 5) | .4 | 0.2 |
| Strong | 4 (2, 4) | 3 (3, 5) | 4 (3, 5) | 3 (2, 5) | 4 (2, 5) | 3 (2, 5) | .14 | .8 |
| Alive | 3 (2, 5) | 3 (2, 4) | 4 (3, 5) | 3 (1, 4) | 3 (2, 5) | 3 (2, 4) | .031 | .9 |
| Fun | 3 (2, 4) | 3 (2, 4) | 4 (3, 5) | 3 (1, 4) | 3 (2, 5) | 3 (2, 4) | .2 | .047 |
| Full | 3 (2, 4) | 3 (2, 4) | 4 (3, 4) | 2 (1, 4) | 3 (2, 5) | 3 (2, 4) | .4 | .5 |
| Energizing | 3 (2, 3) | 3 (2, 4) | 3 (2, 4) | 3 (1, 4) | 3 (1, 4) | 3 (2, 5) | > .9 | .14 |
| Exciting | 2 (1, 4) | 3 (2, 4) | 3 (2, 4) | 2 (2, 4) | 3 (2, 4) | 3 (1, 4) | .4 | .026 |
| Negative | 5 (1, 12) | 4 (1, 10) | 2 (0, 6) | 6 (1, 15) | 5 (1, 16) | 3 (0, 9) | .2 | .4 |
| Miserable | 0 (0, 2) | 0 (0, 1) | 0 (0, 1) | 1 (0, 2) | 0 (0, 2) | 0 (0, 1) | .7 | .8 |
| Bad | 0 (0, 1) | 0 (0, 1) | 0 (0, 1) | 0 (0, 1) | 0 (0, 2) | 0 (0, 1) | .3 | .6 |
| Empty | 1 (0, 2) | 1 (0, 1) | 0 (0, 1) | 1 (0, 2) | 1 (0, 2) | 0 (0, 1) | .3 | .6 |
| Lifeless | 0 (0, 1) | 0 (0, 1) | 0 (0, 1) | 0 (0, 2) | 0 (0, 2) | 0 (0, 1) | .2 | .8 |
| Unpleasant | 1 (0, 2) | 1 (0, 1) | 0 (0, 1) | 1 (0, 2) | 1 (0, 1) | 0 (0, 1) | .093 | .2 |
| Dull | 1 (0, 2) | 1 (0, 2) | 1 (0, 1) | 1 (0, 2) | 1 (0, 2) | 1 (0, 2) | .4 | .2 |
| Weak | 1 (0, 2) | 0 (0, 1) | 0 (0, 1) | 1 (0, 2) | 1 (0, 2) | 1 (0, 1) | .6 | .2 |
| Discouraging | 0 (0, 2) | 1 (0, 2) | 0 (0, 1) | 1 (0, 2) | 1 (0, 2) | 0 (0, 2) | .037 | .5 |
| IMS (N) | 70 | 96 | 87 | 60 | 62 | 39 |  |  |
| Total | 93 (83, 105) | 103 (91, 111) | 109 (98, 122) | 99 (87, 111) | 105 (87, 114) | 105 (95, 116) | > .9 | .058 |
| Presence | 22 (19, 26) | 24 (21, 28) | 27 (23, 31) | 24 (19, 27) | 25 (21, 29) | 26 (24, 29) | .2 | .5 |
| Awareness of Self and Others | 37 (33, 43) | 40 (35, 44) | 41 (37, 47) | 39 (35, 43) | 42 (35, 45) | 42 (35, 47) | .9 | .017 |
| Non-judgmental Acceptance | 14 (12, 16) | 15 (14, 17) | 16 (14, 18) | 14 (13, 17) | 16 (13, 17) | 15 (13, 17) | .2 | .002 |
| Non-reactivity | 20 (18, 24) | 22 (19, 25) | 24 (21, 27) | 21 (18, 24) | 23 (19, 26) | 23 (21, 25) | .3 | .028 |
| Compassion Scale (N) | 70 | 100 | 86 | 57 | 62 | 37 |  |  |
| Total | 66 (58, 71) | 66 (60, 70) | 71 (64, 75) | 63 (58, 71) | 65 (59, 71) | 66 (61, 71) | .7 | .13 |
| Indifference | 15 (13, 18) | 16 (14, 17) | 16 (15, 19) | 16 (14, 18) | 15 (13, 17) | 15 (13, 18) | .14 | .057 |
| Kindness | 17 (14, 20) | 16 (15, 19) | 18 (16, 20) | 16 (14, 19) | 17 (14, 19) | 17 (14, 19) | .2 | .3 |
| Mindfulness | 17 (14, 19) | 17 (15, 18) | 18 (16, 20) | 16 (14, 18) | 17 (15, 19) | 18 (16, 20) | .3 | .9 |
| Common Humanity | 17 (15, 19) | 17 (15, 18) | 18 (15, 20) | 16 (15, 18) | 17 (15, 19) | 17 (14, 19) | .6 | .3 |

*N value is variable at each time point due to variable participant activity

**Supplementary Table 4**

*Positive and Negative Relationship Quality scale results at each timepoint*

| Characteristic | Baseline | Post | Week 6 | p-value | effect size*^2^* |
| --- | --- | --- | --- | --- | --- |
|  | N * = 179 | N = 171 | N = 129 |  |  |
| Positive | 21 (16, 31)^1^ | 25 (18, 33) | 28 (21, 36) | <.001 | .12 |
| Enjoyable | 3 (2, 4) | 3 (3, 5) | 4 (3, 5) | .005 | .04 |
| Pleasant | 3 (2, 4) | 3 (3, 5) | 4 (3, 5) | <.001 | .09 |
| Strong | 3 (2, 4) | 3 (3, 5) | 4 (3, 5) | .014 | .04 |
| Alive | 3 (2, 4) | 3 (2, 5) | 4 (3, 5) | .004 | .05 |
| Fun | 3 (2, 4) | 3 (2, 4) | 3 (2, 5) | <.001 | .07 |
| Full | 2 (2, 4) | 3 (2, 4) | 3 (2, 4) | <.001 | .13 |
| Energizing | 2 (2, 4) | 3 (2, 4) | 3 (2, 5) | <.001 | .07 |
| Exciting | 2 (2, 3) | 3 (2, 4) | 3 (2, 4) | <.001 | .09 |
| Negative | 6 (2, 13) | 4 (1, 12) | 2 (0, 7) | <.001 | .08 |
| Miserable | 1 (0, 2) | 0 (0, 2) | 0 (0, 1) | <.001 | .11 |
| Bad | 0 (0, 1) | 0 (0, 1) | 0 (0, 1) | <.001 | .03 |
| Empty | 1 (0, 2) | 1 (0, 2) | 0 (0, 1) | .003 | .04 |
| Lifeless | 0 (0, 1) | 0 (0, 2) | 0 (0, 1) | .063 | .05 |
| Unpleasant | 1 (0, 2) | 1 (0, 1) | 0 (0, 1) | <.001 | .04 |
| Dull | 1 (0, 2) | 1 (0, 2) | 1 (0, 1) | <.001 | .04 |
| Weak | 1 (0, 2) | 1 (0, 2) | 0 (0, 1) | <.001 | .04 |
| Discouraging | 1 (0, 2) | 1 (0, 2) | 0 (0, 1) | .007 | .07 |

*N value is variable at each time point due to variable participant activity

*^1^* Median (IQR)

*^2^* Partial eta square

**Supplementary Table 5**

Correlations among changes in outcomes

|  | 1 | 2 | 3 | 4 | 5 | 6 |
| --- | --- | --- | --- | --- | --- | --- |
| 1.Change in IMS total | - |  |  |  |  |  |
| 2.Change in Positive PN-RQ | .48^**^ | - |  |  |  |  |
| 3.Change in Negative PN-RQ | -.16 | -.53^**^ | - |  |  |  |
| 4.Change in Social relationship Flourish scale | .34^**^ | .55^**^ | -.36^**^ | - |  |  |
| 5.Change in PSS total score | -.48^**^ | -.41^**^ | .33^**^ | -.51^**^ | - |  |
| 6.Change in compassion total score | .59^**^ | .50^**^ | -.27* | .33^**^ | -.28^**^ | - |

^*^p<.05. ^**^p<.01.

**Supplementary Fig. 1 PN-RQ (Positive components) scores at all timepoints**

**
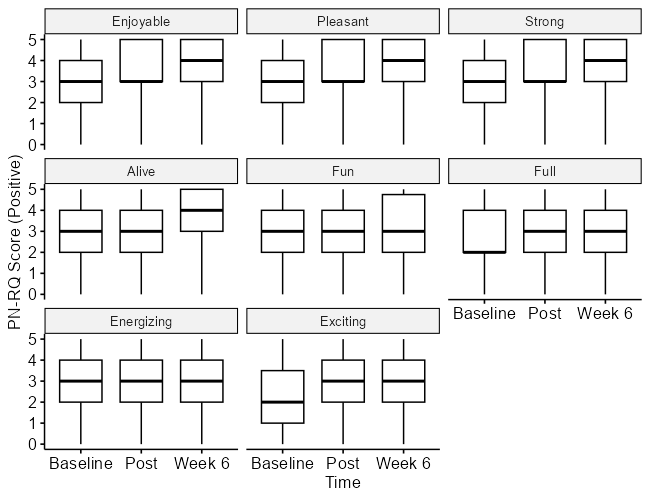
**

**Supplementary Fig. 2 PN-RQ (Negative components) scores at all timepoints**

**
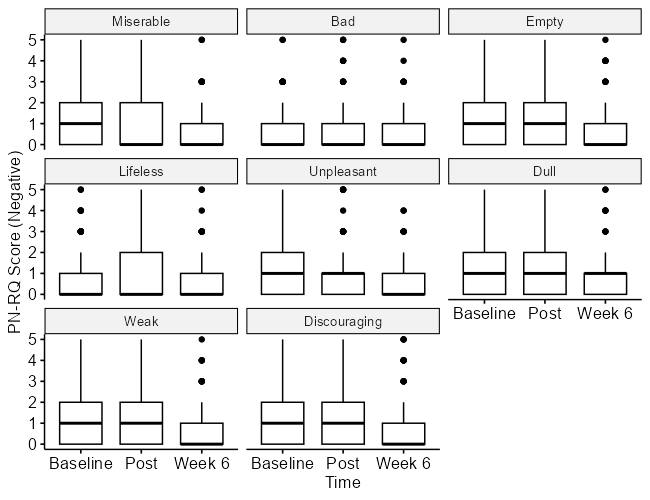
**

**Supplementary Fig. 3 PN-RQ (Positive and Negative) scores at all timepoints by compliance*
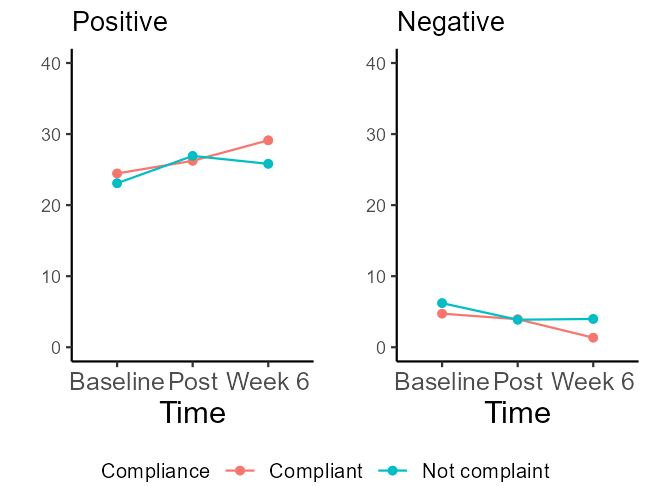
***

**Supplementary Fig. 4 PN-RQ (Positive components) scores at all timepoints by compliance**

**
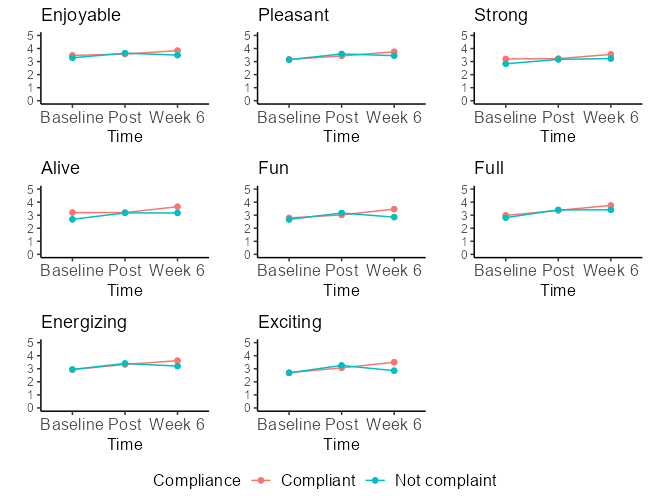
**

**Supplementary Fig. 5 PN-RQ (Negative components) scores at all timepoints by compliance**

**
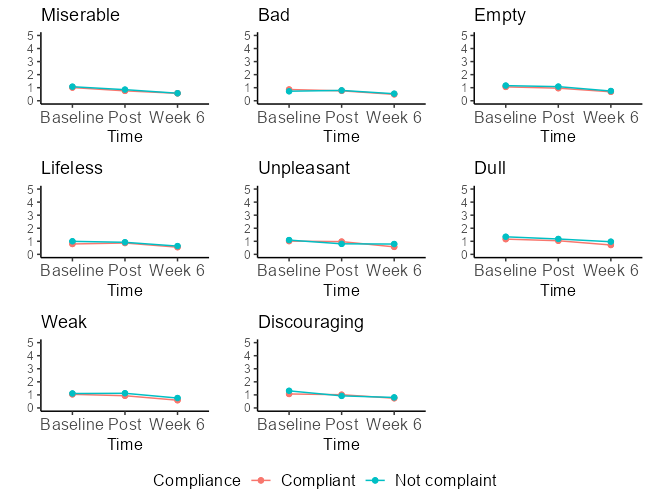
**

**Supplementary Fig. 6 Interpersonal Mindfulness Scale (Components) at all timepoints**

**
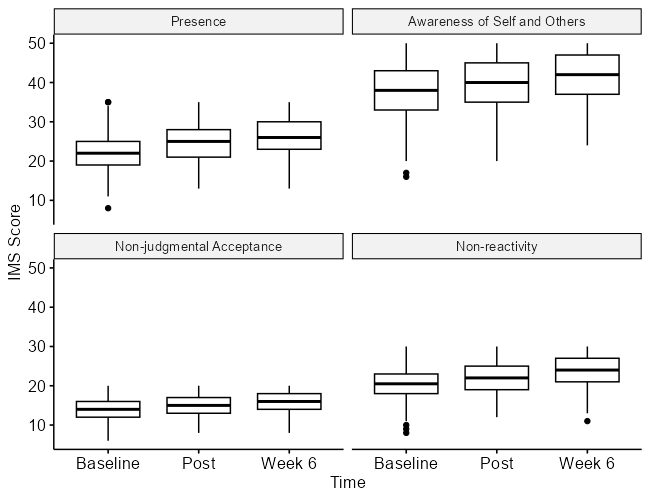
**

**Supplementary Fig. 7 Interpersonal Mindfulness Scale (Components) at all timepoints by compliance**

**
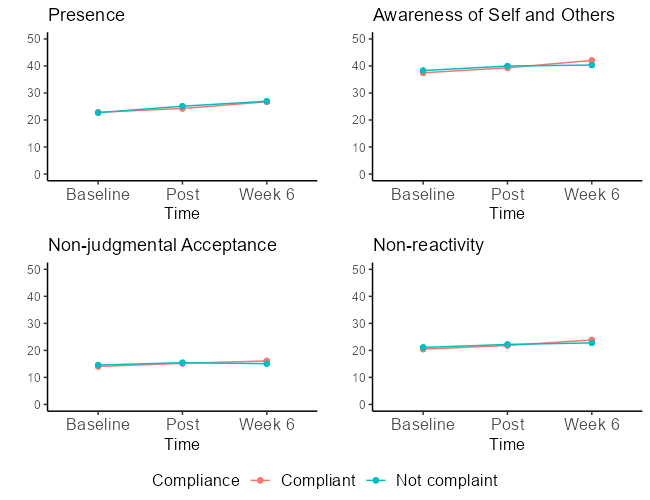
**

**Supplementary Fig. 8 Compassion scale (subscales) at all timepoints**

**
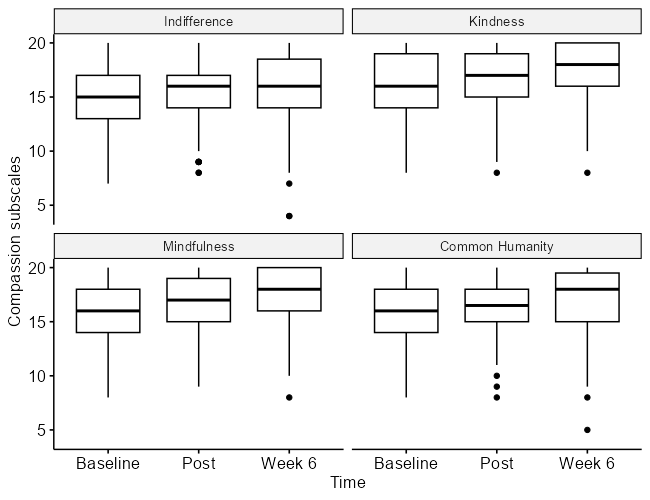
**

**Supplementary Fig. 9 Compassion scale (subscales) at all timepoints compliance**

**
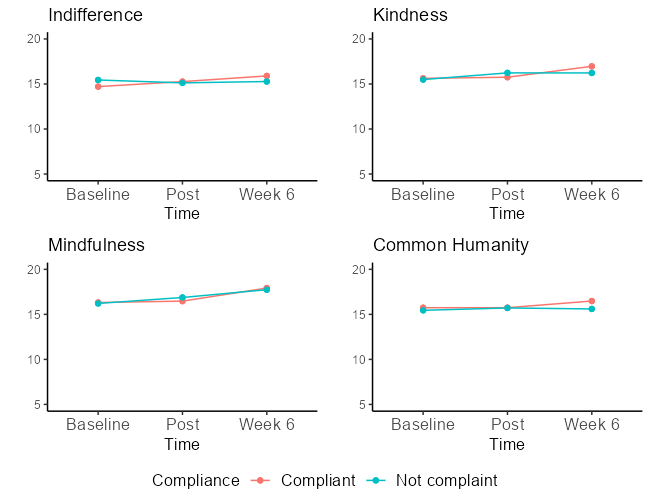
**

**Supplementary Fig. 10 PSS score at all timepoint by compliance**

**
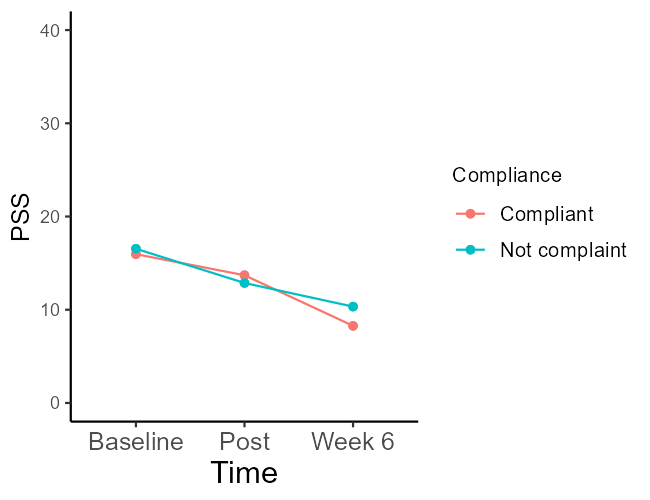
**

**Supplementary Fig. 11 Flourishing Scale at all timepoints (based on compliance)**


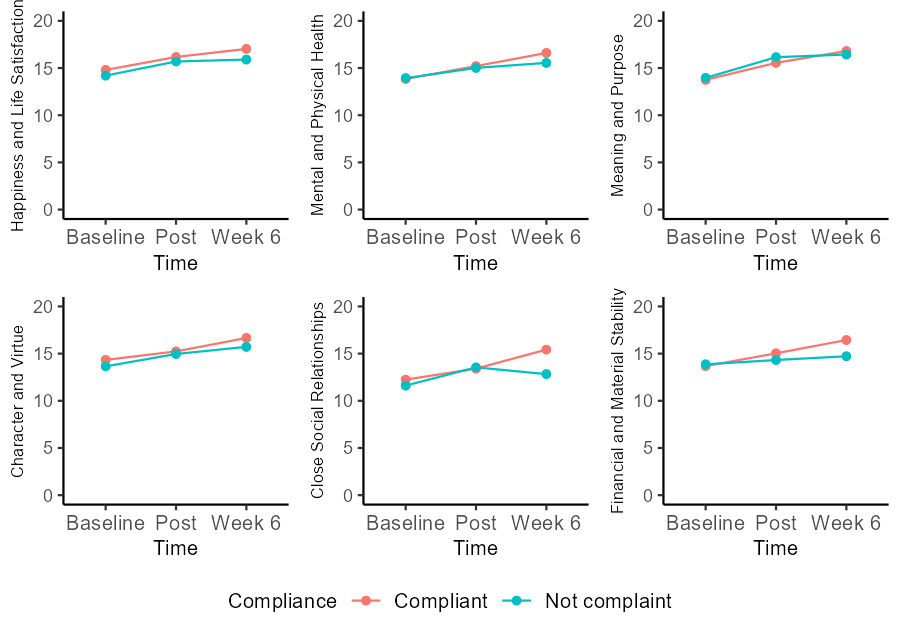

Supplement: Supplementary file 1 [file Table_1.docx]
